# Patient safety incidents are common in primary care: A national prospective active incident reporting survey

**DOI:** 10.1371/journal.pone.0165455

**Published:** 2017-02-14

**Authors:** Philippe Michel, Jean Brami, Marc Chanelière, Marion Kret, Anne Mosnier, Isabelle Dupie, Anouk Haeringer-Cholet, Maud Keriel-Gascou, Claire Maradan, Frédéric Villebrun, Meredith Makeham, Jean-Luc Quenon

**Affiliations:** 1 Comité de coordination de l’évaluation et de la qualité en Aquitaine, Bordeaux, France; 2 Hospices Civils de Lyon and Univ. Lyon, Université Claude Bernard Lyon 1, HESPER, Lyon, France; 3 Haute Autorité de santé, Saint Denis, France; 4 Département de médecine générale, Université Lyon I, Lyon, France; 5 Open Rome et réseau des Grog, Paris, France; 6 Médecin généraliste, Paris, France; 7 RéQua, Réseau qualité de Franche-Comté, Besançon, France; 8 Augustines' clinic, Malestroit, France; 9 Centres municipaux de santé, Saint-Denis, France; 10 Australian Institute of Health Innovation, Macquarie University, Sydney, New South Wales, Australia; Institute of Tropical Medicine (NEKKEN), Nagasaki University, JAPAN

## Abstract

**Background:**

The study objectives were to describe the incidence and the nature of patient safety incidents (PSIs) in primary care general practice settings, and to explore the association between these incidents and practice or organizational characteristics.

**Methods:**

GPs, randomly selected from a national influenza surveillance network (n = 800) across France, prospectively reported any incidents observed each day over a one-week period between May and July 2013. An incident was an event or circumstance that could have resulted, or did result, in harm to a patient, which the GP would not wish to recur. Primary outcome was the incidence of PSIs which was determined by counting reports per total number of patient encounters. Reports were categorized using existing taxonomies. The association with practice and organizational characteristics was calculated using a negative binomial regression model.

**Results:**

127 GPs (participation rate 79%) reported 317 incidents of which 270 were deemed to be a posteriori judged preventable, among 12,348 encounters. 77% had no consequences for the patient. The incidence of reported PSIs was 26 per 1000 patient encounters per week (95% CI [23‰ -28‰]). Incidents were three times more frequently related to the organization of healthcare than to knowledge and skills of health professionals, and especially to the workflow in the GPs’ offices and to the communication between providers and with patients. Among GP characteristics, three were related with an increased incidence in the final multivariable model: length of consultation higher than 15 minutes, method of receiving radiological results (by fax compared to paper or email), and being in a multidisciplinary clinic compared with sole practitioners.

**Conclusions:**

Patient safety incidents (PSIs) occurred in mean once every two days in the sampled GPs and 2% of them were associated with a definite possibility for harm. Studying the association between organizational features of general practices and PSIs remains a major challenge and one of the most important issues for safety in primary care.

## Introduction

Adverse event surveys, set up in hospital settings in developed and developing countries using large representative samples [1,2], have showed convergent results: around 10% of the inpatients experiment an adverse event during their stay and that rate does not appear to decrease with time, apart from a recent exception in the Netherlands [3]. In primary care, studies of PSIs, less numerous than in hospitals, have been set up in the USA [4–7], England [8,9], Australia [10], Germany [11], Canada [12], Israel [13], the Netherlands [14] or France [15,16]. They provide heterogeneous results [17] for a number of reasons. The national and international public health and patient safety agenda is still largely focused on hospitals [18] [19]. Secondly, the concept of harm is heterogeneous because the level of harm caused by PSIs in primary care is less severe than within hospitals, and several harm definitions have been used. In addition, the organizational structure of primary health care in many countries (including France) is one where each primary care practice (and especially general practice) works as an independent business with its own organizational culture and dynamic. Finally, a limited amount of information held in practice systems hinders the use of the medical record review method in primary care [20, 21]. In France, no national representative data on patient safety in primary care was available despite the number of encounters with patients being much higher in primary care than in hospitals (one million GPs consultations per day compared with approximately 400,000 hospital inpatients per day [22]).

In 2013, the first national program for patient safety was launched with four components (engaging and empowering patients for patient safety; training and educating for patient safety; generalizing reporting and learning systems of adverse events; and developing research on patient safety). The French Ministry of Health immediately mandated a group of researchers to undertake a national survey with the aim of measuring PSIs in the primary care general practice setting, in order to have an initial evaluation of patient safety in primary care. The aims were to investigate the nature of reported PSIs, their frequency of being reported and the association between the incidence of reported PSIs and characteristics of the participating GPs’ practice and organization.

## Materials and methods

The research team was an expert group of eight academic GPs with a special interest in patient safety and of two epidemiologists. The ESPRIT study (Etude nationale en Soins PRImaires sur les événemenTs indésirables—national primary care study on PSIs) was an incidence study with PSIs collection by GPs participating in an influenza surveillance network (GROG Network). The study monitored incidents reported on a voluntary and anonymised basis in a random sample of general practices [23].

### Definition of incident

An incident was defined following a two-stage process, including focus groups and consensus among primary care experts and GPs’ representatives, as "an event or circumstance that could have resulted, or did result, in harm to a patient, and which should not be repeated again”. This process has been previously described [24]. The definition of PSI is similar to that used previously by other research in primary care, including the WHO [25], the US Applied Strategies for Improving Patient Safety (ASIPS) [6], and an international pilot study of primary care error reporting involving six countries [26]. This definition encourages reporters to focus on a posteriori judged preventable incidents that may have an association with patient harm.

In order to classify reported incidents, two taxonomies were selected by the primary care research team, being the TAPS (Threats to Australian Patient Safety Study) version of the International Taxonomy of Medical Error in Primary Care [27], which provides a reproducible and internationally recognized codification structured around the major categories of systems versus knowledge based incidents, and the Tempos classification [28], for its current use in French general practice. This classification describes events in terms of (unsafe) dynamic control of multiple parallel constraints by the GP and is based on a framework integrating five time scales termed 'tempos' requiring parallel tasking and processing by GPs: the disease's tempo (unexpected rapid evolutions, slow reaction to treatment); the office's tempo (day-to-day agenda and interruptions); the patient's tempo (time to express symptoms, compliance, emotion); the system’s tempo (time for appointments, exams, and feedback); and the time to access to knowledge (the doctor’s tempo).

### Sampling

The population was a nationwide network of over 800 primary care physicians from all over mainland France (S1 appendix). They are all general practitioners in activity, and had volunteered to report cases during winter 2013 as members of a sentinel network for influenza surveillance. The sample was constituted of GPs randomly selected from these physicians. They were then invited by telephone to participate in the study. Based on a UK study that had used a similar method, we calculated that 2665 patient encounters was required to measure an estimated 75 PSIs per thousand encounters with a precision of 10 per thousand. [22]. As the average number of encounters per day was estimated to be 20, and the average number of working days was 4.3 days per week, we needed a minimum of 31 GPs. As we anticipated a 50% response rate amongst invited GPs, our conservative estimate was that a minimum sample of 100 physicians would be needed. We sampled 120 GPs, adjusted by gender to reflect the national distribution (40% female and 60% male). If a GP declined to participate or did not respond after six calls, the next GP on the list was called.

The participant GPs were trained on the protocol and the data collection method via a written procedure posted by mail. They were specifically trained to the definition of patient safety incident (PSI): the network coordinator organized individual or collective sessions by phone with each of the participating doctors. A video was available [23]. The main objective was to reach a shared understanding on what constituted an incident, as defined in the previous paragraph.

### Data collection and tools

Over the data collection period, participant GPs completed a daily incident reporting form on a web based tool, which was developed and tested by the research group based on an extensive literature review. Both content and face validity were determined by piloting the reporting form with physicians from the network. The reporting form contained 25 questions of which 10 were open-ended questions for the description of the incidents, their contributing factors and the consequences (no noticeable clinical consequence, temporary or reversible harm, definite possibility for harm), both for the patient and for the GPs office organization (S2 Appendix). In cases where multiple incidents were observed by a GP for the same patient encounter, the GP was asked to report on the PSI that they felt had the most potential for severe harm. Each participant GP chose a specific week within the six week data collection period in May-June 2013 and completed the forms during their working hours. Participating GPs completed a questionnaire detailing their daily count of patient encounters, in terms of office consultations and visits at home or in nursing homes.

Two other questionnaires were completed by the GPs: (i) a GP profile (socio-demographic and professional characteristics–number of continuous professional development sessions followed last year, duration of an encounter, number of encounters in a week) and (ii) a questionnaire on the organization of the office (appointment slots for emergency presentations, existence and type of secretary, formalized protocols for emergent calls, computerized records, electronic prescribing software, decision support tool for management of chronic conditions, use of prescription software alarm, method of receiving laboratory results, method of receiving radiological results, digital archiving processes, resident supervision, and type of exercise).

A secured internet gateway was used to conduct the survey.

### Quality control

During electronic data entry, most of the fields were mandatory to complete in order to move to the next screen, and this avoided information being missed. Real-time checks for coherence of the data entry were conducted (coherence of dates, missing information). A weekly review of all incident reports was undertaken by one member of the expert group. Information that was missing and could potentially be useful for further analysis was determined, and GPs were called back to provide missing detail when needed.

At the end of the data collection, a two-day meeting was organized for the expert group and each incident was reviewed by five pairs of experts, in order to: (i) validate their inclusion (conformity with the definition), (ii) score the preventability (would it not have occurred if the patient had received ordinary standards of care, appropriate at the time of the study), (iii) sort them into the two classifications (TAPS taxonomy and Tempos). In cases of discrepancy, the two reviewers tried to reach a consensus and, failing that, the entire expert group discussed the report in order to assign a consensus classification.

### Reproducibility analysis

The Kappa coefficient (κ) was calculated within each pair of experts for four variables: validation of reports as incidents according to the definition, preventability, classification according to TAPS taxonomy [26] (at the level 3, most detailed descriptor) and to the Tempo’s method (principal tempo among the five defined above) [28].

### Data analysis

Incidence of reported PSIs was defined as the ratio of the number of encounters with at least one PSI and the total number of encounters included during the study period. The frequency of incidents and of a posteriori judged preventable incidents and the frequency by type of incident were calculated.

The association between the number of incidents and the organizational characteristics was based on a negative binomial regression model, which can be used to model count data with over-dispersion [29]. The deviance test was calculated at each step of the Poisson model in order to verify that over-dispersion was present: if present, the negative binomial regression was used. The number of encounters was an offset variable. Dependent variables were all variables from the organizational questionnaire and the practice characteristics of the physicians (supervision of residents and isolated or grouped practice). Discrete variables were used for continuous variables (length of GPs consultations, annual number of continuous professional development full-day and late-afternoon sessions), because the linear assumption was not met. Modeling was done according to the Hosmer-Lemeshow procedure, by stepwise increments with a threshold significance of 0.25 in univariable analysis for inclusion of variables, and stepwise decrements with a threshold significance of 0.05 for exclusion of variables in multivariable analysis.

### Ethical aspects

The participating doctors provided their written informed consent to participate by email. Ethical approvals of the study were given by the Ile-de-France III committee and by the CNIL (French data protection authority).

## Results

### GPs main characteristics

**Fig 1:**
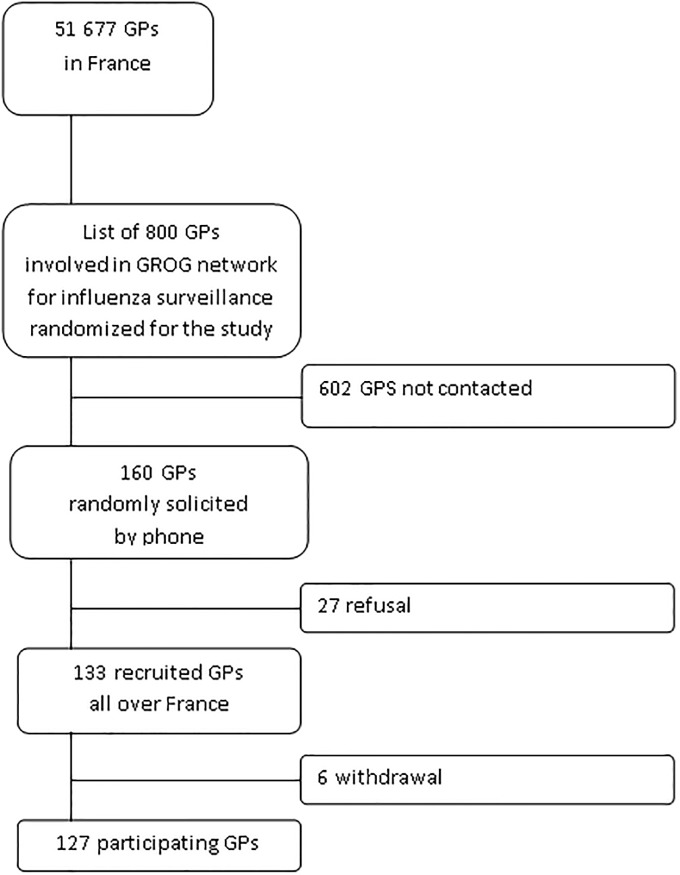
Flowchart of GPs recruitment.

Out of the 160 GPs who were contacted, 27 (17%) refused to participate (Fig 1). Subsequently, out of the 133 included in the study, six (5%) withdrew during the data collection. Thus, 127 GPs participated (79%) for a total of 649 days; 51 (40%) were female and 76 male, working in 19 out of the 22 French regions. The selected GPs mean age was 54 years, 3 years older than that of the French GPs population; they worked more frequently in a group (60% versus 48% respectively) and in rural areas (17% versus 15% respectively); 60% of the respondents were in charge of supervising residents, which is at odds with the proportion of French GPs population in charge of residents (only 15%) (Fig 1).

### Incidence, type and consequences of incidents

Over the reporting period, there was a total of 12,348 encounters (office visits and home visits), and 694 reports made by participant GPs. Almost half of these were ruled out as fitting the study definition of a patient safety incident, mainly because they were problems that could not have resulted in harm to the patient. Therefore a total of 317 PSIs were reported, of which 270 were determined to be preventable. The overall incidence was 26 PSIs per 1000 encounters per week [CI 23‰; 28‰], and 22 a posteriori judged preventable PSIs per 1000 encounters per week [CI 19‰; 24‰]. As the participant GPs had a mean of 20 encounters per day, this corresponded to approximately one PSI being reported every two days for these GPs. The frequency of PSIs related to consultations undertaken outside of the practice and to consultations occurring at the practice were similar (Table 1).

**Table 1:** Incidence for 1 000 patient encounters by type of encounter.

Type of encounters	Nb encounters	All incidents			Preventable incidents		
		number	Incidence (‰)	CI 95%	number	Incidence (‰)	CI 95%
**Office visits**	11,023	276	25‰	[22‰; 28‰]	235	21‰	[19‰; 24‰]
**Home visits**	1,325	41	31‰	[22‰; 40‰]	35	26‰	[18‰; 35‰]
**Total**	12,348	317	26‰	[23‰; 28‰]	270	22‰	[19‰; 24‰]

Out of 317 incidents, 244 (77%) had no noticeable clinical consequence for the patient at the time of data collection; in 21% there was a temporary or reversible harm (for example urinary tract infection in relation to a delay in the treatment; diabetes fainting in relation to delay in test results management) and in 2% a definite possibility for harm (S3 Appendix).

According to classification using the TAPS taxonomy, 204 a posteriori judged preventable incidents (76%) were errors relating to the processes of healthcare (type 1) and 66 (24%) were deficiencies in the knowledge and skills of health professionals (Table 2).

**Table 2:** Incidence for 1 000 encounters according to TAPS taxonomy.

TAPS taxonomy	All incidents			Preventable incidents		
	number	Incidence (‰)	CI 95%	number	Incidence (‰)	CI 95%
**1 Errors related to the processes of healthcare**	**246**	**20‰**	[17‰; 22‰]	**204**	**17‰**	[14‰; 19‰]
**1.1 Errors in practice and healthcare systems**	76	6‰	[5‰; 7‰]	60	5‰	[4‰; 6‰]
**1.2 Investigation errors**	34	3‰	[2‰; 4‰]	31	3‰	[2‰; 3‰]
**1.3 Medication errors**	57	5‰	[3‰; 6‰]	53	4‰	[3‰; 5‰]
**1.4 Treatment errors (non-medication)**	19	2‰	[1‰; 2‰]	15	1‰	[1‰; 2‰]
**1.5 Communication errors and process errors not otherwise specified**	60	5‰	[4‰; 6‰]	45	4‰	[3‰; 5‰]
**2 Errors related to the knowledge and skills of health professionals**	**71**	**6‰**	[4‰; 7‰]	**66**	**5‰**	[4‰; 7‰]
**2.1 Errors in diagnosis**	15	1‰	[1‰; 2‰]	12	1‰	[0‰; 2‰]
**2.2 Errors in managing patient care**	56	5‰	[3‰; 6‰]	54	4‰	[3‰; 6‰]

A principal tempo was defined in 256 out of the 270 a posteriori judged preventable incidents: 44% related to the office's tempo, 24% to the doctor tempo, 18% to the system tempo, 10% to the patient tempo and 3% to the disease tempo (Table 3).

**Table 3:** Incidence for 1 000 encounters according to the tempo method.

Type d’EIAS selon la méthode des tempos	All incidents			Preventable incidents		
	number	Incidence (‰)	CI 95%	number	Incidence (‰)	CI 95%
**Office’s tempo**	126	10‰	[8‰; 12‰]	113	9‰	[7‰; 11‰]
**Doctor’s tempo**	63	5‰	[4‰; 6‰]	61	5‰	[4‰; 6‰]
**Disease’s tempo**	19	2‰	[1‰; 2‰]	9	1‰	[0‰; 1‰]
**Patient’s tempo**	40	3‰	[2‰; 4‰]	27	2‰	[1‰; 3‰]
**System’s tempo**	50	4‰	[3‰; 5‰]	46	4‰	[3‰; 5‰]

The Kappa coefficient between the two experts undertaking the incident validation was 0.54 (25% positive match; 70% consensus after initial disagreement and 5% were arrived at with consultation of the expert group). The concordance was moderate. The reproducibility of preventability assessment was 0.53, 0.45, 0.49 and 0.74 of classification according to TAPS taxonomy at the most detailed level and according to each of the five tempos, respectively.

### Organizational characteristics of GPs offices

Forty percent of the GPs had isolated solo practice, 56% were in group practices, and 4% worked in multi-disciplinary offices. The median number of encounters (office and home visits) during the week was 91 [first and third quartiles (Q1-Q3): 70–119]. The median length of office visits was 17 minutes [Q1-Q3: 15–20 minutes]. Sixty-three percent of GPs were in practices where consultation appointments were not made (patients walked into the clinic and waited for the next available doctor).

The number of continuous professional development sessions undertaken by reporting GPs per year was described in terms of the number of full-day sessions (median 4, Q1-Q3: 1–6) and of late-afternoon (3 hours) sessions (median 8, Q1-Q3: 3–10).

Seventy-nine percent of the GPs had a secretary; either located at the GP’s office (69%), or remotely located via telephone (23%), or both (8%). Among the offices that had a secretary, 79% had formal protocols for emergency presentations. Ninety-six percent used a computerized medical record, 75% systematically used electronic prescribing software with decision support, and 58% used electronic decision support tools for the management of chronic and complex conditions. Thirty-three percent systematically disabled their prescription software alarms. The GPs received pathology results by secure email (71%), postal mail (25%) or by fax (4%), and radiology results by postal mail (72%), by email (26%) and by fax (2%). Sixty one percent of doctors used a digital archive containing their entire records, and 21% with partial records (Table 4).

**Table 4:** Univariate analysis of association between organizational characteristics of GP offices and frequency of incidents.

Dimension	Modalities	Number of respondents	Number	Percentage	Relative risk [95%CI]	p-value
Appointment slots for emergency presentations	Yes	123	45	37%	1.05 [0.74; 1.51]	0.78
	No		78	63%	-	
Secretary	Yes	123	97	79%	1.04 [0.67; 1.61]	0.86
	No		26	21%	-	
Type of secretary	Office	123	67	55%	1.08 [0.68; 1.70]	0.75
	Phone		22	18%	1.01 [0.58; 1.79]	0.96
	Two		8	6%	0.78 [0.35; 1.75]	0.55
	No secretary		26	21%	-	
Formalized protocols for emergent calls	Yes	123	77	63%	1.17 [0.66; 2.07]	0.59
	No		20	16%	1.01 [0.64; 1.57]	0.98
	No secretary		26	21%	-	
Computerized records	Yes		118	96%	0.93 [0.38; 2.27]	0.88
	No		5	4%	-	
Electronic prescribing software	Yes systematically	123	92	75%	1.38 [0.65; 2.90]	0.40
	Yes sometimes		21	17%	1.20 [0.63; 2.31]	0.58
	No		10	8%	-	
Decision support tool for management of chronic conditions	Yes systematically	123	71	58%	1.32 [0.78; 2.26]	0.30
	Yes sometimes		22	18%	1.12 [0.73; 1.70]	0.61
	No		30	24%	-	
Use of prescription software alarm	Yes	123	82	67%	1.23 [0.85; 1.77]	0.28
	No		41	33%	-	
Method of receiving laboratory results	Paper	123	31	25%	-	
	email		87	71%	1.33 [0.89; 1.98]	0.17
	fax		5	4%	2.07 [0.88; 4.87]	0.10
Method of receiving radiological results	Paper	123	89	72%	-	
	Email		32	26%	1.14 [0.77; 1.70]	0.51
	Fax		2	2%	0.64 [0.17; 2.44]	0.52
Digital archiving processes	Yes totally	123	75	61%	1.28 [0.74; 2.22]	0.38
	Yes incompletely		26	21%	0.94 [0.59; 1.49]	0.78
	No		22	18%	-	
Resident supervision	Yes	127	76	60%	1.33 [0.93; 1.90]	0.12
	No		51	40%	-	
Type of exercise	Solo	127	51	40%	-	
	Group of physicians		71	56%	1.13 [0.78; 1.61]	0.52
	Multidisciplinary team		5	4%	2.27 [0.99; 5.22]	0.05

### Links between organizational characteristics and frequency of incidents

The median count of reported PSIs during the week of data collection was three, ranging from zero to 19 (Q1-Q3: 1–5).

During the univariable analysis, five variables were found to be statistically associated with the PSIs incidence with a threshold significance of 0.25 (Table 4). During multivariable analysis, two variables were excluded because they were found to be not statistically significant: number of continuous professional development full-day session attendance and resident supervision. The final model contained three variables (Table 5). The relative risk of reporting a PSI when receiving laboratory results by fax was 2.71 (p<0.02) compared with by paper or email. For GPs in a multidisciplinary primary care clinic compared with sole practitioners, the relative risk of reporting a PSI was 2.26 (p = 0.03). An increase of one minute per patient encounter resulted in a relative risk of reporting a PSI of 1.05 (p = 0.02).

**Table 5:** Estimate of relative risks (final model).

Variable	Modalities	Relative risk	[95%CI]	p-value
Length of consultation (minute)	<=15 min	-		
	>15 min et <=20 min	1.61	[1.12; 2.31]	**0.01**
	>20 min	1.68	[0.95; 2.97]	0.07
Method of receiving laboratory results	Paper	-		
	Email	1.30	[0.87; 1.92]	0.20
	Fax	2.71	[1.17; 6.27]	**0.02**
Type of exercise	Solo	-		
	Group of physicians	1.10	[0.77; 1.58]	0.60
	Multidisciplinary team	2.78	[1.25; 6.20]	**0.01**

## Discussion

### Key results

GPs reported 26 PSIs per 1000 encounters or a mean of one PSI every two days in their practice with a large variability between GPs. Incidents were three times more frequently related to the organization of healthcare than to knowledge and skills of health professionals, and especially to the workflow in the GPs office and to the communication between providers and patients. Their very diverse nature is illustrated by examples in S3 Appendix.

### Limitations

More than half of the 694 incidents reported by GPs were deemed not to be PSIs according to the study’s definition after review by the expert group. This suggests that the level of understanding of the definition of PSI by participant GPs was not high, despite the time and effort that was spent in preparation of the study participants before with patient safety concepts (focus groups, video recording, e-learning). This issue has been described in previous studies [30], and should be given particular attention by researchers who try to set up a primary care reporting system for PSIs.

There was a large proportion of PSIs reported (244 out of 317, i.e., 77%) which did not have long-term consequences for the patient, as found in other primary care research [14,31]. These incidents are less threatening as they present less medico-legal risk for participants to report than incidents which caused harm to the patient. In addition, the most frequent type of PSI found in claims-based studies, delayed diagnoses, is missed: they usually escape the short term and are difficult to identify by GPs in their daily practice [7, 15]. Hence, it is worth asking whether in fact more serious PSIs in terms of patient harm were under-represented.

There was an unexpectedly large range of reported PSIs per participant. The number of incident reports made by individual GPs varied between 0 and 19 reports, despite the methods of collection being identical and the sample of doctors chosen from a population of GPs who were all familiar with data collection tasks. This may reflect different levels of awareness, a factor that may explain two of the three associations found in this study between organizational factors and level of reporting.

The kappa statistics of the review process for PSIs and preventability assessment were modest. To our knowledge, there is no data in the literature on the reproducibility of such process regarding PSIs in primary care but a large literature for adverse event (AE) detection with record review in hospital settings. The latest review showed a moderate inter-rater reliability for AE assessment (κ = 0.40–0.57) in 10 studies, although the inter-rater reliability was substantial (κ = 0.61–0.80) in four others; the κ for all studies assessing preventable AEs ranged from 0.19 to 0.76. All methods use an implicit review style, meaning that the AE assessment relies on expert judgment instead of using well defined criteria on a checklist [32]. Our results on the reproducibility of the classification into the two taxonomies were similar to the previous studies: the concordance amongst the coders in the validation study of the TAPS taxonomy was 0.66 [27], and the one in the Brami’s tempo study was 0.68 [28].

A limitation of our study findings is that we did not investigate patient factors that were associated with PSIs. Previous literature investigating adverse events in primary care from a large UK study has shown that factors such as older patient age and an increased number of co-morbidities has been associated with an increased risk of adverse events, and being known for longer by a practice with a reduced risk [33]. As we did not collect this information, we are unable to comment on patient factors that may have contributed to PSIs in our data.

### Generalizability

The study participants were not necessarily representative of French GPs as they were already involved in an influenza surveillance network to report incidents. Studies using random samples of GPs in France are frequently impaired by low participation rates; usually less than 50% agree to participate [34]. We thought that a sample of randomly-chosen physicians within a population that was potentially biased towards reporting was preferable to a sample from the whole GP population in order to improve the likelihood of having PSIs reported. Moreover, the quality of data collection was key as we needed sufficient information on the PSIs, their causes and consequences for the secondary assessment of the PSIs by experts. The population used for our sample may have contributed to an optimization of the quality of data collection, without this necessarily affecting the results: there is no indication that GPs engaged in flu surveillance are any more aware of practice and organizational issues in relation to patient safety. On the other hand, we cannot rule out that this sample of GPs was more concerned by collective and organizational issues, including patient safety. We provided a strong supportive infrastructure with training, online entry, real-time coherence tests during data entry and weekly assessment of the reports by an expert. Other research has recently been undertaken that tries to improve on these methodological issues, however the reporting frequency was not increased: despite a supportive infrastructure, 124 voluntary respondents only made 264 reports in 36 months (<1 report per person per year) [32]. Therefore we reduced the data collection period to a brief time period (a week).

This selection bias being discussed, we believe that the generalizability is acceptable, as the results are based on over 12,000 observations from a randomly selected sample with over 100 clusters and a robust data collection process.

### Interpretation

By extrapolation, our results suggests that in the fiscal year 2013 (one million GPs encounters/day), between 23,000 and 28,000 PSIs occurred, of which between 18,000 and 21,500 were without long-term consequence for the patient. This is a total of between 19,000 and 24,000 a posteriori judged preventable incidents per year. But in terms of disability or death, 520 (2%) could have serious consequences.

These results are in the same range as the ones of another large observational French study [16] (ECOGEN), conducted in 2012, which is however not representative of the situation in France. In this study, the frequency of AE was estimated at one per GP per day. The incidence was also in the range of the other international large-scale, prospective study based on a similar incident definition [6, 9, 12, 35, 36, 37, 38, 39]. With regard to the tempos classification, our results were different from those that were previously published [28], which were extracted from patients' insurance claims.

The organizational factors in Primary Care may be key to explain the variations found in the frequency of reported PSIs per participant. Longer consultation lengths may be interpreted in different ways: GPs are more likely to detect incidents when they take more time. GPs with an older patient clientele, with multi-morbidity and polypharmacy, may spend more time with their patients. GPs with longer consultation lengths presumably deal with more care processes and therefore experience difficulties (prioritization challenges, memory or attention problems, etc.) which can ultimately result in patient safety problems [12,40]. Lastly, GPs facing organizational problems may spend more time to solve it. Regarding the factor “type of exercise”, there is evidence in the primary care literature supporting the inclusion of multidisciplinary teams to improve the disclosure of adverse events [41]. Any hypothesis for explaining the third factor (receiving laboratory results by fax compared with by paper or email) would be hazardous. This association may be related to a third factor that was not measured, or is related with multiple testing. In addition, the number of physician receiving laboratory results by fax was low (5), reflecting a rare situation which will in addition disappear in the next years.

These findings confirm that the use of PSI reports is insufficient to determine the true underlying proportions of different types of patient safety threats occurring at a population level in Primary Care [42, 43]. Yet, to date, incident reporting remains the most common method; actually the only one affordable at reasonable cost, for counting and classifying PSIs in primary care settings [44]. Record review is not feasible and less appropriate because the information system is not mature and because primary care does not operate in time-limited events like a hospital admission and records may be dispersed across many provider. One potential way forward to improve the knowledge content of studies based on reporting systems would be to undertake more qualitative work on the data [45].

There is a growing call for patient safety incident reporting to move from being a research tool to being embedded in Primary Care health systems. Zwart et al explored the feasibility of a local incident reporting system as a tool for safety management and organizational learning in five primary healthcare centers: they showed a greater uptake of incident reporting by primary care clinicians when reports were dealt with by local groups of ‘experts’ and the local clinicians were involved in designing improvement efforts [36,46]. The issue of increasing PSI reporting is challenging because of the considerable fragmentation of the workforce and the far less structure in its organization compared to hospitals, compounded by a lack of clear governance and patient safety leadership [47]. In France, multidisciplinary mortality and morbidity review meetings have commenced in Primary Care [48]. This remains to be evaluated, along with the role of PSI reporting systems in increasing safety culture and behavior changes and ultimately making healthcare safer for patients outside of the hospital setting.

### Conclusions

The sheer size of patients passing through our primary care systems on a daily basis makes the absolute numbers of patients being avoidably harmed a highly significant issue for improvement in this health care setting. Studying the association between organizational features of GPs and PSIs remains a major challenge. However this is probably one of the most important issues for safety in primary care.

## Supporting information

S1 Database Negative binomial analysis. (TXT)

S1 Appendix Brief description of the French health care system. (DOCX)

S2 Appendix Patient safety incident questionnaire. (DOCX)

S3 Appendix Summary examples of patient safety incident reports. (DOCX)
