# Supplementary material for: Patient safety incidents are common in primary care: A national prospective active incident reporting survey
Source: PLoS One. 2017 Feb 14;12(2):e0165455. doi: 10.1371/journal.pone.0165455 (PMC5308773; doi:10.1371/journal.pone.0165455)
Supplement: S1 Appendix — (DOCX) [file pone.0165455.s002.docx]

S1 Appendix 1: Brief description of the French health care system

The French health care system consists of employed doctors in hospitals (public and private sector) and independent practitioners who have their own ambulatory care practice. As of 1st January 2014, the total number of doctors in France was 215 539. There were 130 295 independent practitioners and 51 677 general practitioners. Overall, there were 281.4 practitioners (GPs and specialists) per 100,000 inhabitants.

GPs provide medical consultations in their office or during house calls. It is common for specialists to have both a private consultancy and work part-time in a hospital.

The other independent health care professionals (such as nurses, physical therapists etc) total over 96,000.

*See : Atlas de la démographie médicale en France. Situation au 1er janvier 2015. Conseil national de l’ordre des médecins. http://www.conseil-national.medecin.fr/sites/default/files/atlas_national_de_la_demographie_medicale_2015.pdf*
