# Supplementary material for: Patient safety incidents are common in primary care: A national prospective active incident reporting survey
Source: PLoS One. 2017 Feb 14;12(2):e0165455. doi: 10.1371/journal.pone.0165455 (PMC5308773; doi:10.1371/journal.pone.0165455)
Supplement: S2 Appendix — (DOCX) [file pone.0165455.s003.docx]

S2 Appendix: Patient Safety Incident Questionnaire

**Q1.** Does the safety incident affect a patient in particular? Yes No

**Q2**. What is your level of knowledge of this patient?

I know him very little I know him little I know him well I know him very well

**Q3**. What is his year of birth?

**Q4**. What is his sex? Female Male

**Q5.** Are there any communication problems between the patient and the practitioner (ie language problem)? YesNo

**Q6**. Which is the socio-professional group of this patient?

Farmer Craftsman, shopkeeper and company director Liberal profession, executive, intellectual and artistic profession Intermediate profession, technician Employee Worker Retired Unemployed person who has never worked Diverse non active (other than retired persons) Pupil, student

**Q7**. What happened? Describe in few words the safety incident.

**Q8**. What is the harm (the consequence) for the patient?

**Q9**. Which is the level of harm?

Death or life-threatening Definitive physical incapacity Temporary physical incapacity No clinical consequence

**Q10.**

GP office Patient home Patient home hospitalisation system Patient home with nursing home service Nursing home Public place Other

**Q11**. When did the safety incident occur?

**Q12**. By whom the safety incident has it been detected?

You Other doctor Pharmacist Other healthcare professional Patient himself People around the patient (close relation, carer…)

**Q13**. When has it been detected?

**Q14**. Describe all the reasons that led to this safety incident (Ask yourself many times the question “Why?”)

**Q15**. What is, according to you, the main cause that led to this safety incident?

**Q16**. Is the safety incident related to your office organization? Yes No

**Q17**. May the safety incident be related to a possible lack in your competence or a misuse of your skills? Yes No

**Q18**. Is the safety incident related to an unusual progression of the disease? Yes No

**Q19**. Is the safety incident related to a specific characteristic of the patient? Yes No

**Q20**. Is the safety incident related to a dysfunction in the coordination of care? Yes No

**Q21**. Who has been involved in the occurrence of this safety incident?

**Q22**. What are the consequences of this safety incident on your office (known and potential)?

**Q23**. At which frequency do you think you meet this safety incident in your practice?

First time Rarely (1-2 times per year) Sometimes (3-11 times per year) Frequently (>1 time per month)

**Q24**. What would have been able to prevent the causes?

**Q25**. Other comment
