# Supplementary material for: Patient safety incidents are common in primary care: A national prospective active incident reporting survey
Source: PLoS One. 2017 Feb 14;12(2):e0165455. doi: 10.1371/journal.pone.0165455 (PMC5308773; doi:10.1371/journal.pone.0165455)
Supplement: S3 Appendix — (DOCX) [file pone.0165455.s004.docx]

**S3 Appendix: Summary examples of patient safety incident reports**

**a – Classified using the TAPS Taxonomy** [25]

**1. Errors related to the processes of healthcare**

*1.1. Errors in practice and healthcare systems*

The office secretary informs the GP that one of his patients (82 year-old female patient), living in a nursing home, has fractured her femoral neck. She gives him the phone number to call the patient's husband, still living at home. Due to another patient at the practice having a similar name, the GP mistakenly calls the husband of the other patient, who is very distressed by the call.

*1.2. Investigation errors*

A 47 year-old patient was treated for colon cancer two years ago. Due to being occupied with managing acute cardio-vascular symptoms for the past year, the GP forgot to undertake the needed quarterly cancer surveillance check-up. This led to an increased risk of a diagnostic delay in detecting cancer recurrence.

*1.3. Medication errors*

At the end of a consultation with a known male patient, his accompanying wife, 61 years old, asked the doctor to examine her painful ear. Due to external otitis, GP decided to prescribe local quinolone treatment but forgot to write “ear drops”. As patient was prone to tendinitis pharmacist called him back to check the prescription.

*1.4 Treatment errors (non-medication)*

During the pneumococcal vaccination of a 2 year-old child, he moved unexpectedly which dislodged the needle tip, and it was estimated that half of the vaccine dose was not administered correctly. This led to a risk of insufficient vaccine protection.

*1.5 Communication errors and process errors not otherwise specified*

A 22 year-old female patient presented with iron deficiency anaemia due to heavy menstrual bleeding. She was prescribed iron supplementation, but not informed by the GP of the iron treatment effect on her stool colour. Her concern led to her ceasing the treatment prematurely.

**2 Errors related to the knowledge and skills of health professionals**

*2.1. Errors in diagnosis*

A 14 year-old female patient attended the GP twice in three months reporting that her menstrual period was delayed. The diagnosis of pregnancy was missed at the first consultation, due to the patient reporting that she was not sexually active. The GP had questioned this young patient on that occasion in her mother's presence, and felt that the history taking had therefore been inadequate and resulted in the diagnostic delay.

*2.2 Errors in managing patient care*

An 86 year-old female patient was suffering for high blood pressure. The GP mistakenly prescribed a dosage of anti-hypertensive drugs that was too high for the patient. This resulted in patient harm (bradycardia and a fall with reversible head trauma).

**b – Classified using the Tempos method**

**Office tempo**: see above examples 1.3 and 1.1 from TAPS taxonomy

**Access to knowledge**: see above examples 2.2, 1.4 and 1.5 from TAPS taxonomy

**Disease tempo**: see above example 2.1 from TAPS taxonomy

**Patient tempo**

A 73 year-old male patient with cardiovascular disease, treated with balanced anticoagulation therapy, presented a light accidental fall, with a subdural hematoma requesting surgery.

**Out-office coordination/referral tempo**.

A 69 year-old diabetic female patient Called the practice to obtain the results of a urine microscopy and culture pathology test. An error in result checking caused her to be given an incorrect result by phone that her culture was negative, as an interim report was used by mistake. This delayed the discovery that she had a positive bacterial culture on her final result, and led to delayed treatment of her urine infection which resulted in uncontrolled diabetes.

**c –Serious patient harm and potential harm**

Example 1:

An 87 year-old male patient with chronic cardiac failure and anticoagulant treatment was seen for prescription renewal. The patient was having intermittent breathing difficulties. The patient had been intending to address this with his usual cardiologist, but when he attempted to make an appointment several weeks earlier, had been unable to get in to his usual cardiologist for a month. The patient was reviewed the next day by another cardiologist who sent him to hospital. The patient died during hospitalisation with cardiac pulmonary oedema. The reporting GP believed that appropriate care had been delayed due to the usual cardiologist not being available.

*TAPS taxonomy: 1.1. Errors in practice and healthcare systems*

*Tempo method: Out-office coordination/referral tempo*

Example 2:

A 77 year-old male patient was having anticoagulant treatment following a recent cerebro-vascular accident (stroke). His anti-coagulant blood results indicated that his dosage needed urgent treatment adjustment, but the patient was not able to be contacted due to a wrong phone number having been recorded in his electronic health record. This resulted in an increased risk of stroke.

*TAPS taxonomy: 1.1. Errors in practice and healthcare systems*

*Tempo method: Office tempo*
